# Endophytic *Alternaria alternata* Culture-Derived Elicitor Promotes Growth and Antibacterial Activity in *Kalanchoe laetivirens*

**DOI:** 10.1021/acsomega.5c06091

**Published:** 2025-08-05

**Authors:** Khaoanny de Souza, Nelson Barros Colauto, Gabrielle Caroline Peiter, Renato Eising, Patricia Dayane Carvalho Schaker

**Affiliations:** † Graduate Program in Bioscience Technologies (PPGBio), Federal Technological University of Paraná (UTFPR), Toledo, Paraná 85902-490, Brazil; ‡ Federal Technological University of Paraná (UTFPR), Graduate Program in Chemical and Biotechnological Processes (PPGQB), Toledo, Paraná 85902-490, Brazil

## Abstract

*Kalanchoe* spp. are valued for their antioxidant and antimicrobial properties, primarily attributed to bioactive compounds like phenolic acids. Endophytic fungi can stimulate compound production, growth, and microbial control in tissue culture. This study aims to assess the presence of selected phenolic compounds, the growth-promoting effects, and antibacterial activity of *in vitro*-cultivated *Kalanchoe laetivirens* plantlets supplemented with a powdered fungal elicitor derived from a nonliving culture. The elicitor was obtained from an antioxidant-producing endophytic fungus, previously isolated from *K. laetivirens* and cultured on Murashige-Skoog medium. Among 16 endophytic fungi tested via DPPH• (2,2-diphenyl-1-picrylhydrazyl) assay, *Alternaria alternata* (strain 16) showed the highest antioxidant activity. Identified through microscopic and ITS rDNA sequencing, this strain was cultured, powdered, and used as an elicitor in Murashige-Skoog medium for *K. laetivirens* explants. Extracts from elicited and nonelicited *in vitro*-cultivated plantlets and nonelicited *in vivo*-cultivated plantlets were analyzed by high-performance liquid chromatography for phenolic compounds and tested for antibacterial activity using the microdilution method. Ferulic acid was detected only in *in vitro*-cultivated plantlets, *p*-coumaric acid was present under all conditions, and other phenolic compounds were absent in all cases. Elicited *in vitro* plantlets showed the most potent antibacterial activity against *Escherichia coli* and *Staphylococcus aureus*. A 1:1 ratio of *p*-coumaric to ferulic acids resulted in the highest antibacterial efficacy, while an unbalanced 2:1 ratio reduced activity. These results suggest that *in vitro* elicitation improves growth and enhances the antibacterial profile of *K. laetivirens*, offering a strategy to enhance bioactive compound production and potentially manage systemic, especially nonculturable, phytopathogens.

## Graphical abstract

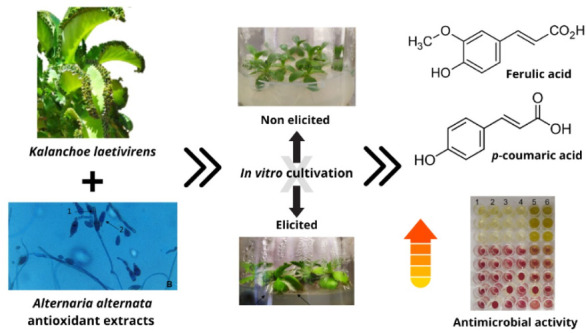

## 1 Introduction

Microorganisms that live symbiotically within plant tissues—referred to as endophytes—play a pivotal role in modulating host metabolism and stimulating the biosynthesis of bioactive secondary metabolites with therapeutic and ecological relevance.^1^ Among these metabolites, phenolic compounds stand out for their involvement in plant defense mechanisms against biotic and abiotic stressors.^2^ However, the biosynthesis of these compounds is highly variable and affected by multiple factors, including the developmental stage pf the plant, environmental conditions (e.g., light intensity, temperature, and humidity), and the composition of the plant’s endophytic microbiota.^3^

To address the challenges posed by this natural variability, plant tissue culture techniques, such as clonal propagation and regeneration, offer a promising alternative by providing controlled and reproducible environments for secondary metabolite production.^2^ These *in vitro* systems facilitate the standardization of biosynthetic process and provide a platform for applying specific stimuli to enhance metabolite accumulation. In this context, elicitation—the application of chemical or biological agents to trigger secondary metabolism—has emerged as an effective strategy to amplify the production of key phytochemicals under laboratory conditions.^4^

One promising approach to elicitation involves the use of endophytes as biotic elicitors in plant tissue cultures. Endophytes have been reported to stimulate metabolite biosynthesis, improve explant development, and reduce microbial contamination.^5,6^ For instance, antioxidant-producing endophytic fungi isolated from *Kalanchoe* spp. (excluding *Kalanchoe laetivirens*) have been reported to induce the accumulation of phenolic compounds, such as ferulic acid, which contribute to plant growth and antimicrobial defense.^7^ However, the use of live microorganisms in tissue cultures carries certain risks, such as disrupting morphogenetic processes due to uncontrolled microbial proliferation. To overcome this, nonviable powdered microbial elicitors, derived from processed endophytic cultures, have been proposed as safer and more stable alternatives. These preparations retain bioactive properties and can be incorporated into culture media, replicating the beneficial effects of endophytic microbiota without compromising the stability of the culture system.

Given the promising results observed with other *Kalanchoe* spp., attention has increasingly turned to *K. laetivirens*, a species known for its rich phytochemical profile and medicinal properties. *Kalanchoe* spp. are widely recognized for their antioxidant and antimicrobial activities,^8^ which are attributed to the presence of phenolic acids and related compounds.^9^ Among these, ferulic acid stands out for its potent antioxidant properties and its potential for applications in various biotechnological fields.^7,10^ Phytochemical studies have identified various secondary metabolites in *K. laetivirens*, including phenolic compounds, flavonols, flavones, flavononols, flavanones, xanthones, and alkaloids.^11^ Despite this diversity, its biotechnological potential remains underexplored, particularly in relation to metabolite induction via microbial elicitation.^12,13^

To address this gap, the present study investigates the presence of selected phenolic compounds (*p*-coumaric acid, ferulic acid, gallic acid, catechin, chlorogenic acid, and quercetin), along with the growth-promoting effects and antibacterial activity of *K. laetivirens* plantlets cultivated *in vitro*. The plantlets were supplemented with a powdered elicitor derived from a nonviable culture of an antioxidant-producing endophytic fungus previously isolated from *K. laetivirens*. This fungal biomass was incorporated into Murashige-Skoog medium to assess its ability to stimulate secondary metabolism and enhance biological activity. The results contribute to advancing our understanding of microbial elicitation in plant systems and support the development of sustainable biotechnological strategies for enhancing plant-derived antimicrobial compounds and improving phytopathogen management.

## 2 Materials and Methods

### 2.1 Biological Material

Endophytic fungi were isolated from the roots and stems of *Kalanchoe laetivirens* Desc. (Crassulaceae), a specimen collected at the Federal University of Technology – Paraná (UTFPR), Toledo campus, in Toledo, Paraná, Brazil (24°43′57’“S, 53°45′52”’ W). The plant material was taxonomically identified by Temponi L. G., and a voucher specimen was deposited at the Herbarium of the State University of Western Paraná (UNOP from UNOESTE) under the accession number UNOP 12937.

The fungal strains were preserved in mineral oil in the UTFPR–Toledo culture collection. For experimental purposes, all isolates were revived, subcultured on potato dextrose agar (PDA; 39 g L^–1^), and incubated at 28 ± 1 °C in the dark. The recovered isolates were then used as inoculum for subsequent assays.

The bacterial strains *Escherichia coli* (Migula) Castellani and Chalmers (ATCC 25922; INCQS 00033) and *Staphylococcus aureus* subsp. *aureus* Rosenbach (ATCC 25923; INCQS 00015) were obtained from the American Type Culture Collection through the National Institute for Quality Control in Health (INCQS) at the Oswaldo Cruz Foundation (FIOCRUZ), Rio de Janeiro, Brazil.

### 2.2 Antioxidant Activity of Fungal Culture Medium

Each strain was cultivated in 250 mL Erlenmeyer flasks containing 150 mL yeast-malt liquid medium (HiMedia) at 28 ± 1 °C with continuous agitation at 150 rpm for 7 days. The inoculum consisted of three PDA discs (0.5 cm in diameter) containing mycelium from each culture. After incubation, the culture medium of each endophyte was vacuum-filtered using filter paper (Whatman Grade 1), and the filtrate was stored at −20 °C for further analysis.

The antioxidant activity of the filtrate was determined using the 2,2-diphenyl-1-picrylhydrazyl (DPPH•) radical scavenging assay, following the method described by Çayan et al.^14^ A methanolic DPPH• solution (0.047 mg mL^–1^) was prepared to achieve an absorbance of approximately 0.95 at 517 nm. Then, 100 μL of filtrate was transferred to a 15 mL Falcon tube, protected from light, and mixed with 3.0 mL of methanolic DPPH• solution. The mixture was vortexed for 3 s (Phenix AP56) and incubated for 25 min. Absorbance was then measured using a UV–vis spectrophotometer (Kasuaki-IL-0082 100). The strain showing the highest antioxidant activity was selected for further investigation.

### 2.3 Fungal Identification

The fungal strain with the highest antioxidant activity was selected for identification and cultured on PDA medium at 28 ± 1 °C under dark conditions. Identification was carried out using a combination of conventional and molecular approaches. Conventional methods included microscopic examination of spore morphology, size, and hyphal structure, following the protocols described by Muniz et al.^15^ and Barroso et al.^16^ Molecular identification was also performed to confirm the taxonomic classification of the strain.

For molecular identification, the fungal strain’s genomic DNA was extracted using the Wizard Genomic DNA Purification Kit (Promega). The polymerase chain reaction (PCR) for amplifying the ITS1–5.8S-ITS2 region of rDNA was performed in a total volume of 50 μL, containing 1x 10x-buffer, 1.5 mM MgCl_2_, 0.2 μM of each primer (ITS1: 5’TCCGTAGGTGAACCTGCGG3′ and ITS4: 5’TCCTCCGCTTATTGATATGC3′), 0.2 mM dNTPs, 0.2 U Taq DNA polymerase (Synapse), and 25 ng DNA. Amplification was carried out in a thermocycler (Thermo Fisher) with the following cycling conditions: initial denaturation at 94 °C for 3 min, followed by 35 cycles of denaturation at 95 °C for 30 s, annealing at 55 °C for 30 s, extension at 72 °C for 1 min, and a final extension at 72 °C for 10 min. The amplification products were visualized on a 1% agarose gel stained with SYBR Safe (Invitrogen). The amplified products were purified using the Wizard SV Gel and PCR Cleanup System (Promega) and sequenced by the Sanger method. The resulting electropherograms were analyzed using BioEdit software^17^ to obtain the consensus sequence. Nucleotide sequences were compared with those in the National Center for Biotechnology Information (NCBI) *nr* database using the Nucleotide BLAST tool, considering coverage, identity, and *E*-value. The sequence was deposited in GenBank.

### 2.4 Elicitor Preparation and Application for *K. laetivirens* Cultivation

To prepare the elicitor derived from a nonliving culture, the selected fungal strain was cultured in 500 mL Erlenmeyer flasks containing 300 mL of potato dextrose liquid medium for 7 days at 28 ± 1 °C and 150 rpm. The inoculum consisted of three PDA discs (0.5 cm in diameter) with mycelial growth of the strain. After cultivation, the fungal culture medium was autoclaved (121 °C for 30 min), oven-dried at 65 ± 2 °C, and ground using a mortar and pestle to obtain a uniform powder. This powder, hereafter referred to as the elicitor, was incorporated into the Murashige-Skoog basal medium (pH 5.8) containing agar (8 g L^–1^, Kasvi). The culture medium was prepared without phytoregulators or an exogenous carbon source. Each 500 mL glass jar contained 200 mL of Murashige-Skoog medium, either supplemented with 10 mg mL^–1^ of the elicitor (treatment) or without it (control).

Shoots of *K. laetivirens* were disinfected using a modified protocol based on Rajamanikyam et al.^18^ and subsequently used as explants. The explants were washed in 70% ethanol for 1 min, then immersed in sodium hypochlorite solution (20 g L^–1^) for 10 min, followed by five rinses with distilled, autoclaved water (121 °C for 20 min). After drying on sterile filter paper in a laminar flow hood, ten explants were inoculated into glass jars containing Murashige-Skoog medium. Five jars were supplemented with the elicitor, while the remaining five served as controls without it. The cultures were maintained for 12 days under controlled conditions with a 16-h light and 8-h dark photoperiod.

### 2.5 Extracts of *K. laetivirens* Plantlets

After the *in vitro*-cultivation period, *K. laetivirens* plantlets were oven-dried with air circulation at 65 ± 2 °C until a constant mass was achieved. The dried plantlets were then ground using a knife mill, and 1 g of each sample was extracted with 90% ethanol for 30 min in an ultrasound bath, according to Souza et al.^19^ The extracts were vacuum-filtered using filter paper (Whatman grade 1), and the resulting filtrates were stored in amber bottles. The *in vivo*-cultivated plantlets were processed using the same protocol as the *in vitro*-cultivated plantlets.

### 2.6 Characterization of Phenolic Compounds from *K. laetivirens* Plantlets

The plantlet extracts (3 mg mL^– 1^) were analyzed for phenolic compounds using high-performance liquid chromatography (HPLC) according to Çayan et al.,^14^ with modifications. The analysis was conducted on an HPLC (Dionex UltiMate 3000-Thermo Fisher Scientific) equipped with a UV–vis detector (VWD-3400RS), quaternary pump (LPG-3400SD), column oven (TCC-3400RS), and a manual injection system (20 μL). Detection was conducted at 220 nm, with additional monitoring at 210, 282, and 203 nm. Chromatographic separation was achieved using a ZORBAX Eclipse XDB C18 column (250 mm × 4.6 mm, 5 μm) maintained at 40 ± 1 °C. The mobile phase consisted of (A) ultrapure water acidified with acetic acid (5 mL L^–1^; pH 3.03) and (B) methanol acidified with acetic acid (5 mL L^–1^), with a flow rate of 0.8 mL min^–1^. Standard solutions of *p*-coumaric acid, ferulic acid, gallic acid, catechin, chlorogenic acid, and quercetin were prepared at a concentration of 1 mg mL^–1^ to support compound identification. These standards were analyzed under the same chromatographic conditions used for the plantlet extracts during the method development phase. The retention times obtained from the standards served as reference values for reliable identification of the corresponding peaks in the experimental chromatograms.

### 2.7 Antibacterial Activity of *K. laetivirens* Plantlet Extracts

A sterile 96-well round-bottom (U-shaped) microplate was prepared for each bacterial strain (*E. coli* and *S. aureus*). Each well was filled with 100 μL of Mueller-Hinton broth, followed by the addition of 100 μL of plantlet extract to the first well of each column at the following initial concentrations: 150 mg mL^–1^ for the nonelicited *in vitro*-cultivated plantlets, 93 mg mL^–1^ for the elicited *in vitro*-cultivated plantlets, and 100 mg mL^–1^ for the nonelicited *in vivo*-cultivated plantlets. After homogenization, a 100 μL aliquot was sequentially transferred across the column in a 2-fold serial dilution (1:1, 1:2, 1:4, 1:8, 1:16, 1:32, 1:64, and 1:128), generating final concentrations of 75, 37.5, 18.75, 9.37, 4.68, 2.34, 1.17, and 0.58 mg mL^–1^ for nonelicited *in vitro*-cultivated plantlets; 46.5, 23.25, 11.62, 5.81, 2.9, 1.45, 0.75, and 0.36 mg mL^–1^ for elicited *in vitro*-cultivated plantlets; and 50, 25, 12.5, 6.25, 3.12, 1.56, 0.78, and 0.39 mg mL^–1^ for nonelicited *in vivo*-cultivated plantlets. The initial extract concentrations were selected based on the maximum soluble concentration achievable for each extract type, which reflected variations in extraction yield, phytochemical composition, and physicochemical properties. This approach ensured the biological relevance of each preparation and allowed for a consistent and comparable assessment of antibacterial potency across all plantlet groups.

A 10 μL aliquot of bacterial inoculum, standardized to 0.1 on the McFarland scale, was added to all wells except the blank wells, which received 10 μL of saline solution (9 g L^–1^). The negative control consisted of Mueller–Hinton broth, the same vehicle used for extract dilutions, and the bacterial inoculum. The microplates were incubated at 35 ± 1 °C for 22 h, after which 20 μL of TTC solution (5 g L^–1^; 2,3,5-triphenyltetrazolium chloride) was added to each well. The microplates were incubated for another 2 h to allow colorimetric visualization of bacterial viability.^20^ The minimum inhibitory concentration (MIC) was determined to be the lowest extract concentration, which completely inhibited microbial growth. All assays were performed in duplicate.

### 2.8 Statistical Analysis

Antimicrobial assays were performed in triplicate for each microorganism, and the results were expressed as the arithmetic mean ± standard deviation. Data were analyzed using one-way analysis of variance (ANOVA), followed by Tukey’s honestly significant difference (HSD) test at a significance level of α = 0.05.

## 3 Results

Sixteen strains were successfully recovered on PDA medium from 30 endophytic fungi preserved in mineral oil, isolated from the roots and stems of *K. laetivirens*. The recovered strains were then cultivated in the yeast-malt liquid medium, and their antioxidant activity was analyzed (Figure 1). Strains 4 and 16 exhibited the highest antioxidant activities of 76 and 80%, respectively, with strain 16 selected for further analysis.

**Figure 1:**
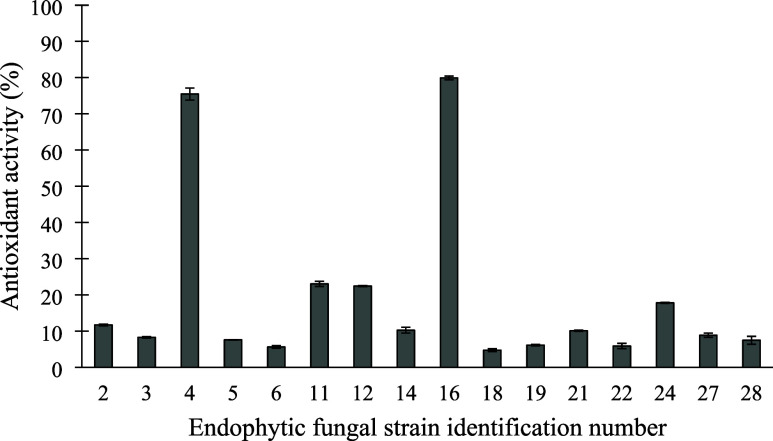
Antioxidant activity of endophytic fungal strains from *Kalanchoe laetivirens* cultivated in yeast-malt liquid medium, assessed using the DPPH• (2,2-diphenyl-1-picrylhydrazyl) method.

Strain 16 was identified as *Alternaria alternata* (Fr.) Keissl. based on light microscopy analysis, exhibiting characteristic morphological features described by Muniz et al.,^15^ such as inverted pear-shaped, ovoid, or ellipsoid conidia with both transverse and longitudinal septations (Figure 2A,B). Additionally, the conidiophores were simple, straight or curved, smooth, containing one to three septa, and terminating in an apical perforation (Figure 2B). The conidia formed long, branched chains, a defining feature of *A. alternata* (Figure 2A), consistent with the description by Barroso et al.^16^

**Figure 2:**
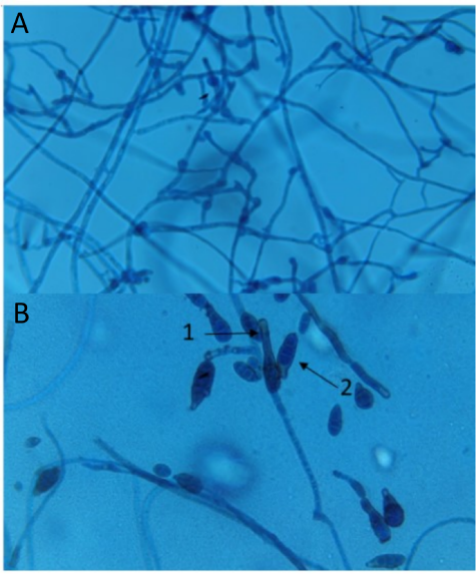
Mycelium and spores of the endophytic fungus identified as *Alternaria alternata*, isolated from *Kalanchoe laetivirens* (strain 16), observed under light microscopy: (A) hyphae at 400× magnification; (B) characteristic *A. alternata* spores (conidia) at 1000× magnification, showing a short beak (arrow 1), measuring one-third the length of the conidia body, and a transverse septum (arrow 2).

Molecular identification using a 505 bp ITS1-ITS4 region amplicon confirmed the fungal strain as *A. alternata*, exhibiting a 98.8% sequence identity with *A. alternata* sequences available in GenBank. This high degree of homology reinforces the accuracy of the morphological identification based on light microscopy analysis, ensuring taxonomic reliability. The amplicon sequence has been deposited in GenBank under accession number OP998049.1.

Elicited and nonelicited *in vitro*-cultivated plantlets exhibited well-developed roots and shoots in the Murashige-Skoog medium (Figure 3A,B). Particularly in the elicited *in vitro*-cultivated plantlets, there was significant development of adventitious roots emerging from stems, along with larger leaves and more robust growth (Figure 3B) than the nonelicited *in vitro*-cultivated plantlets (Figure 3A) and nonelicited *in vivo*-cultivated plantlets (Figure 3C). This indicates that the elicitor promoted the overall growth of the plantlets, particularly by enhancing the development of adventitious roots and larger leaves.

**Figure 3:**
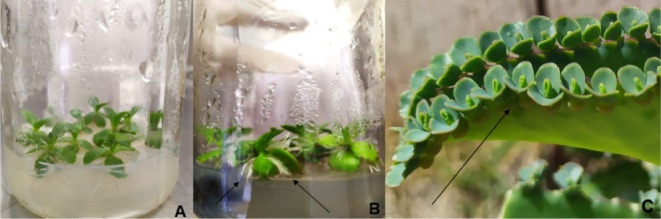
Nonelicited (A) and elicited (B) *in vitro*-cultivated *Kalanchoe laetivirens* plantlets—supplemented with a powdered endophytic *Alternaria alternata* (strain 16) culture-derived elicitor—highlighting adventitious root formation (B, arrows), and nonelicited *in vivo* cultivation of *K. laetivirens* plantlets (C, arrow).

HPLC analysis of ethanolic extracts showed that ferulic acid (retention time: 16.932 min) was detected exclusively in *in vitro*-cultivated plantlets, regardless of elicitation, whereas *p*-coumaric acid (retention time: 15.637 min) was present under all tested conditions (Figure 4). This consistent presence of *p*-coumaric acid suggests that its production is independent of elicitation or cultivation conditions, while the presence of ferulic acid appears to be induced explicitly under *in vitro* cultivation. However, previous studies have reported the natural occurrence of ferulic acid in various plants grown *in vivo*,^10^ indicating that although its induction in *K. laetivirens* occurs primarily under *in vitro* cultivation conditions, it can also be produced *in vivo* under specific environmental or physiological conditions that remain unidentified. Gallic acid, catechin, chlorogenic acid, and quercetin were absent in all cultivation conditions (Figure 4), indicating that the elicitation strategies employed were insufficient to induce their biosynthesis.

**Figure 4:**
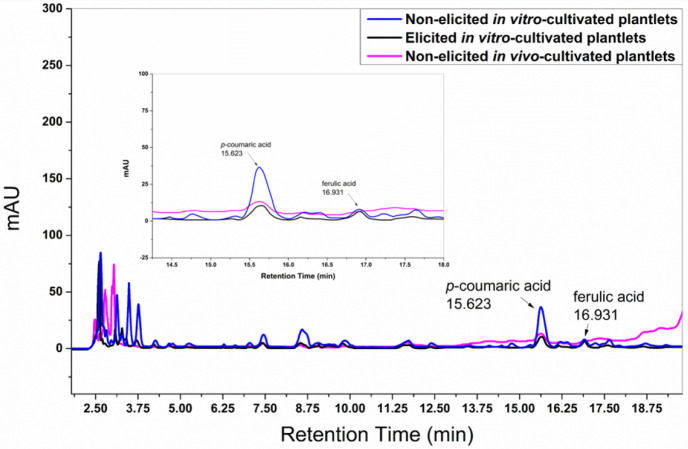
High-performance liquid chromatography analysis of *p*-coumaric acid (retention time = 15.623 min) and ferulic acid (retention time = 16.931 min) in ethanolic extracts from elicited and nonelicited *in vitro*-cultivated plantlets and nonelicited *in vivo*-cultivated plantlets of *Kalanchoe laetivirens* was performed. Compound identification was based on retention time matching with authentic standards previously analyzed under identical chromatographic conditions.

The ethanolic extracts of *K. laetivirens* plantlets demonstrated antibacterial activity against *E. coli* and *S. aureus* under all cultivated conditions (Table 1). Among these, the extract from elicited *in vitro*-cultivated plantlets showed the most effective antibacterial activity, with MIC values of 11.6 mg mL^–1^ against *E. coli* and *S. aureus* (Table 1). The nonelicited *in vivo*-cultivated plantlets produced the second most effective extract, with MIC values of 12.5 against *E. coli* and 25.5 mg mL^–1^ against *S. aureus* (Table 1). In contrast, the extract from nonelicited *in vitro*-cultivated plantlets showed the least effective antibacterial activity, with MIC values of 18.8 and 37.5 mg mL^–1^ against *E. coli* and *S. aureus*, respectively (Table 1). These findings suggest that elicitation during *in vitro* cultivation enhances antibacterial activity, making it a promising strategy for improving plant-derived antibacterial agents.

**Table 1:** Antibacterial Activity of Ethanolic Extracts from Elicited *In Vitro*-Cultivated Plantlets, Non-elicited *In Vitro*-Cultivated Plantlets, and Non-elicited *In Vivo*-Cultivated Plantlets of *Kalanchoe laetivirens* against *Escherichia coli* and *Staphylococcus aureus*

	Minimum inhibitory concentration (mg mL^–1^)	
Ethanolic extract	*Escherichia coli*	*Staphylococcus aureus*
Elicited *in vitro*-cultivated plantlets	11.62 ± 0.40 a	11.62 ± 0.40 a
Nonelicited *in vitro*-cultivated plantlets	18.75 ± 0.75 c	37.50 ± 1.20 c
Nonelicited *in vivo*-cultivated plantlets	12.50 ± 0.40 b	25.00 ± 0.80 b

Values represent arithmetic mean ± standard deviation of three independent replicates. Different letters within each column indicate significant differences among treatments (one-way ANOVA, *p* ≤ 0.05, followed by Tukey’s HSD test).

The highest antibacterial activity was observed in extracts from elicited *in vitro*-cultivated plantlets, suggesting that the combination of *p*-coumaric acid and ferulic acid (Figure 4)—both well-known antimicrobial compounds^6^—contributes synergistically to this effect (Table 1). In contrast, the second-highest antibacterial activity was detected in extracts from nonelicited *in vivo*-cultivated plantlets (Table 1), where ferulic acid was absent, but *p*-coumaric acid was present at a similar level to that found in the elicited *in vitro*-cultivated plantlets (Figure 4). This indicates that *p*-coumaric acid alone accounts for a substantial portion of the observed antimicrobial activity.

Conversely, nonelicited *in vitro*-cultivated plantlets exhibited the lowest antibacterial activity (Table 1), despite containing ferulic acid at the same level as the elicited *in vitro*-cultivated plantlets and *p*-coumaric acid in approximately double the amount found in the other conditions (Figure 4). This result suggests that an unbalanced ratio—with a *p*-coumaric acid to ferulic acid ratio of approximately 2:1—may lead to an antagonistic interaction, reducing the overall antibacterial efficacy.

These findings underscore the importance of a balanced composition of ferulic and *p*-coumaric acids in maximizing antimicrobial potential. While *p*-coumaric acid alone exhibits intermediate antibacterial activity, its combination with ferulic acid in an approximately 1:1 ratio appears to enhance the overall effect, likely through a synergistic interaction. At the same time, the results highlight the complex interplay between phenolic compounds, suggesting that their relative proportions, rather than mere presence, play a critical role in determining the antibacterial efficacy of bioactive *K. laetivirens* extracts.

## 4 Discussion

Strain 16, identified as *A. alternata*, exhibited the highest antioxidant activity among the tested isolates. The antibacterial properties of this fungus have been attributed to pyranones,^21,22^ which also exhibit significant antioxidant potential, as reported by their DPPH• scavenging activity (56.3 μg mL^–1^).^23^ Additionally, *A. alternata* extracellular enzymes are known for extracting ferulic acid from wheat and triticale brans, a phenolic compound widely distributed in plant cell walls with antioxidant properties.^24^ Depending on environmental conditions and host susceptibility, this fungus is frequently isolated from plant tissues, which can function as an endophyte and/or a pathogen.^25^ Its capacity to produce diverse secondary metabolites suggests a potential role in modulating antioxidant and antimicrobial, potentially through phenolic acids like ferulic acid, which enhance plant defense and stress tolerance.^26^ Further investigations are needed to elucidate the specific metabolic pathways involved and determine whether *A. alternata* actively promotes phenolic compound biosynthesis in associated plant systems.

*p*-Coumaric acid was consistently detected across all *K. laetivirens* plantlets cultivation conditions in our study, indicating that it is a characteristic metabolite of the species. This widely distributed phenolic compound in fruits, grains, medicinal herbs, and certain fungi exhibits a broad spectrum of pharmacological activities, including antibacterial properties.^27^ It also plays an essential role in plant defense, enhancing resistance in sweet fruits^28^ and inducing postharvest defense responses against *Alternaria* rot in jujube fruits.^29^ Although *p*-coumaric acid is primarily regarded as a plant-derived defense compound against microbial threats, its detection in endophytic *A. alternata* extracts from other plants, such as *Catharanthus roseus*,^30^ suggests a potential fungal contribution to its biosynthesis. In *K. laetivirens*, its presence is most likely a result of intrinsic plant metabolic activity. However, the involvement of *A. alternata* cannot be entirely ruled out; therefore, further metabolomic and genetic analyses are required to clarify the biosynthetic origins of *p*-coumaric acid in this system.

Ferulic acid was detected in elicited and nonelicited *in vitro*-cultivated plantlets. However, it was absent in nonelicited *in vivo*-cultivated plantlets, suggesting that its biosynthesis is preferentially induced under *in vitro* cultivation conditions. Furthermore, the enhanced antibacterial activity observed in extracts from elicited *in vitro*-cultivated plantlets, particularly against *S. aureus*, indicates that elicitation further stimulates ferulic acid production. Ferulic acid, a major cinnamic acid derivative,^31^ is widely recognized for its antioxidant, anticancer, and anti-inflammatory properties,^32^ exhibiting greater antioxidant potential than many of its derivatives.^33,34^ Beyond its pharmacological significance, ferulic acid plays an essential role in plant physiology by contributing to antioxidant and antimicrobial defense mechanisms.

Although *p*-coumaric acid and ferulic acid were identified in the plantlet extracts, and their relative distribution aligned with the observed antibacterial activity across treatment groups, precise quantification using calibration curves and direct testing of isolated standards at matched concentrations was beyond the scope of this study. Nonetheless, the comparative approach employed—based on well-characterized extracts and consistent biological responses—proved effective in demonstrating the effect of phenolic composition on antibacterial activity. Future studies may build upon these findings by conducting targeted quantification and testing of pure compounds, both individually and in combination, to further elucidate their specific roles and potential synergistic effects.

In our study, elicited *in vitro*-cultivated plantlets exhibited enhanced growth, developing adventitious roots and larger leaves than other cultivation conditions. Adventitious roots, which develop in response to environmental stress,^35^ are associated with increased secondary metabolite production, making them valuable for biotechnological applications.^36^ The observed growth promotion suggests that elicitation enhances secondary metabolite biosynthesis and overall plant development, reinforcing its potential for commercial biotechnological exploitation. This finding is supported by previous reports indicating that endophytic *A. alternata* in *Asclepias sinaica* can promote plant growth by producing ammonia, indole-3-acetic acid (IAA), and other phytohormones.^26^ Such evidence strengthens the hypothesis that fungal elicitors can mimic the effects of endophytes, replicating growth-promoting outcomes through indirect biochemical signaling. In addition to *p*-coumaric and ferulic acids, other phenolic acids such as caffeic, syringic, and protocatechuic acids have been identified in species of the *Kalanchoe* genus,^7^ and may also contribute to the antioxidant activity observed in our study. Although the elicitor effect of *A. alternata* was demonstrated, the specific fungal metabolites responsible for this response were not identified, as LC-MS or HPLC profiling of the fungal culture was not conducted. Future studies should include comprehensive chemical characterization of the fungal biomass to identify and quantify these metabolites, which could help clarify their roles in modulating the antioxidant and antibacterial responses observed in *K. laetivirens*.

Few studies have specifically investigated the antimicrobial activity of *K. laetivirens*,^37,38^ the *Kalanchoe* genus is known for its broad-spectrum antimicrobial properties against bacterial and fungal pathogens.^39^ The limited research on *K. laetivirens* highlights a gap in knowledge and underscores the need for further investigation. Our findings demonstrate that stimulating the biosynthesis of a bioactive compound, such as ferulic acid, through *in vitro* elicitation constitutes a strategy for producing the metabolite under controlled conditions, potentially enhancing antimicrobial efficacy. Thus, integrating *in vitro* cultivation with a powdered fungal elicitor derived from a nonliving culture could be a promising approach to enhance ferulic acid accumulation in *K. laetivirens* plantlets, with potential applications in disease management and plant biotechnology.

Moreover, this elicitor-based stimulation strategy represents a potential application of our findings, which could be further explored through *in vivo* extrapolation to enhance the production of antimicrobial metabolites for the control of systemic phytopathogens, such as *Candidatus Liberibacter asiaticus* (Las), the causal agent of huanglongbing (citrus greening disease), a devastating threat to citrus production.^40^ More broadly, this strategy could be adapted to diverse phytopathogen systems, offering a potentially sustainable and biotechnologically relevant tool for agriculture and plant protection.

## 5 Conclusion

Among all endophytic strains isolated from *K. laetivirens*, strain 16, identified as *A. alternata*, exhibits the highest antioxidant activity. The application of a powdered fungal elicitor derived from a nonliving culture under *in vitro* conditions enhances adventitious root formation and promotes the development of larger leaves in *K. laetivirens*. Ferulic acid is detected exclusively in *in vitro*-cultivated plantlets, while *p*-coumaric acid is present in all cultivation conditions. Gallic acid, catechin, chlorogenic acid, and quercetin are absent in all cultivation conditions. All plantlet extracts exhibit antibacterial activity, with elicited *in vitro*-cultivated plantlets demonstrating the most potent effect against *E. coli* and *S. aureus*. A balanced composition of *p*-coumaric and ferulic acids is essential to maximize antimicrobial potential, as plantlet extracts show higher efficacy at a 1:1 ratio and reduced activity at a 2:1 ratio. Overall, elicitation under *in vitro* cultivation enhances the antibacterial activity of *K. laetivirens*, offering a promising approach for bacterial growth control.
